# Supplementary material for: Flanker: a tool for comparative genomics of gene flanking regions
Source: Microb Genom. 2021 Sep 24;7(9):000634. doi: 10.1099/mgen.0.000634 (PMC8715433; doi:10.1099/mgen.0.000634)
Supplement: Supplementary material 1 [file mgen-7-0634-s001.pdf]

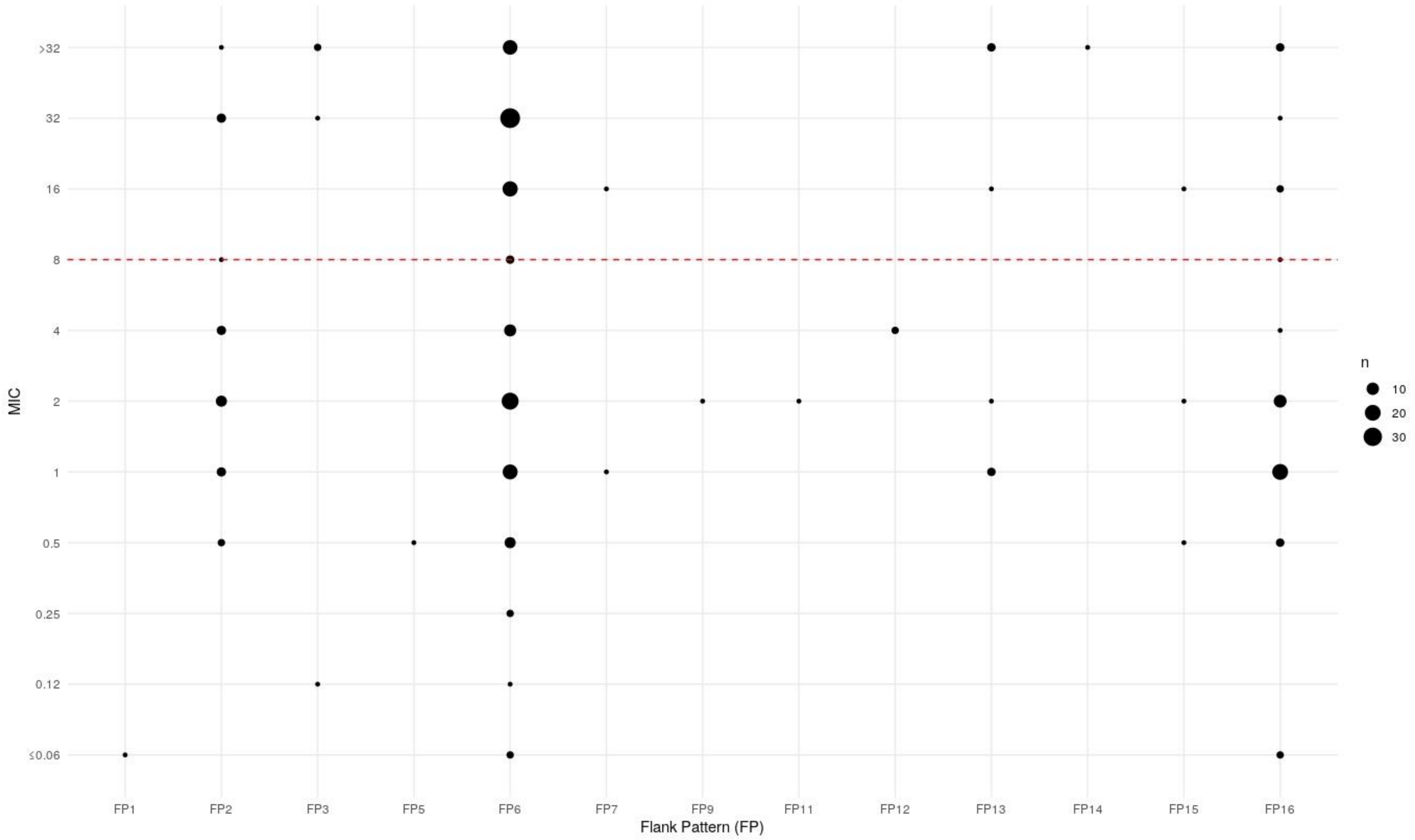
MIC
>32
32
16
8
4
2
1
0.5
0.25
0.12
≤0.06
FP1
FP2
FP3
FP5
FP6
FP7
FP9
FP11
FP12
FP13
FP14
FP15
FP16
Flank Pattern (FP)
n
10
20
30

Figure S1: Distribution of meropenem minimum inhibitory concentrations (MIC mg/L) by Flank Pattern (FP). Circle size indicates the number of observations per category and the red hashed line denotes the EUCAST breakpoint (>8mg/L). Isolates from the EuSCAPE dataset (short read only sequencing, n=226 isolates with linkable phenotype data) were assigned to a Flank Pattern based on the top mash containment hit). No isolates were assigned to FPs 4,8 or 17 and the one isolate assigned to FP10 had no linkable phenotypic data.

Table S1 – NCBI project accession numbers for sequencing data used

Description	Project Accession	Number of isolates
Complete plasmid assemblies containing *bla*$_{KPC2/3}$	PRJEB33308 (EuSCAPE)<br><br>ERP118777 (Dutch CPE)	42<br><br>8
Complete plasmid assemblies containing *bla*$_{OXA-48}$	PRJEB33308 (Dutch CPE)<br><br>PRJNA591727 (EuSCAPE)	9<br><br>42
Short read Illumina fastq files for isolates containing *bla*$_{OXA-48}$*	PRJEB10018 (EuSCAPE)	425
Short read Illumina fastq files for isolates containing *bla*$_{KPC-2/3}$*	PRJEB10018 (EuSCAPE)	442

*Full list of accession numbers used and associated metadata is available at https://doi.org/10.6084/m9.figshare.14074250.v1

Table S2 – Flank Pattern (FP), MEFinder and TETyper calls for blaKPC-2/3 flanking regions. The isolate column gives the contig name within the assembly, the next three columns give FP assignations at 500/5000/7200bp upstream of the gene. Columns 5 to 8 give the MEFinder/TETyper calls for the 500bp/5000bp upstream flanking regions whereas the final column gives TETyper calls when TETyper was given the whole plasmid assembly as input.

Contig Name	Flanker 500bp	Flanker 5000bp	Flanker 7200bp	MEFinder 500bp	MEFinder 5000bp	TETyper 500bp	TETyper 5000bp	TETyper Tn4401 whole
CABFYD010000003.1	1	1	1	Tn4401|1|KT378596.1	Tn4401|1|KT378596.1	1-6602|7020-7118|7202-10006	1-2202|7020-7118|7202-10006	Tn4401a
MT560073.1	1	1	1	Tn4401|1|KT378596.1	Tn4401|1|KT378596.1	1-6602|7020-7118|7202-10006	1-2202|7020-7118|7202-10006	Tn4401a
CABFYQ010000002.1	1	1	1	Tn4401|1|KT378596.1	Tn4401|1|KT378596.1	1-6602|7020-7118|7202-10006	1-2202|7020-7118|7202-10006	Tn4401a

Contig Name	Flanker 500bp	Flanker 5000 bp	Flanker 7200 bp	MEFinder 500bp	MEFinder 5000bp	TETyper 500bp	TETyper 5000bp	TETyper Tn4401 whole
CABFZV010000006.1	1	1	1	Tn4401|1|KT378596.1	Tn4401|1|KT378596.1	1-6602|7020-7118|7202-10006	1-2202|7020-7118|7202-10006	Tn4401a
CABGAG010000002.1	1	1	1	Tn4401|1|KT378596.1	Tn4401|1|KT378596.1	1-6602|7020-7118|7202-10006	1-2202|7020-7118|7202-10006	Tn4401a
CABFYI010000002.1	1	1	1	Tn4401|1|KT378596.1	Tn4401|1|KT378596.1	1-6602|7020-7118|7202-10006	1-2202|7020-7118|7202-10006	Tn4401a
CABGAO010000002.1	1	1	1	Tn4401|1|KT378596.1	Tn4401|1|KT378596.1	1-6602|7020-7118|7202-10006	1-2202|7020-7118|7202-10006	Tn4401a

Contig Name	Flanker 500bp	Flanker 5000bp	Flanker 7200bp	MEFinder 500bp	MEFinder 5000bp	TETyper 500bp	TETyper 5000bp	TETyper Tn4401 whole
CABFYF010000002.1	1	1	1	Tn4401|1|KT378596.1	Tn4401|1|KT378596.1	1-6602|7020-7118|7202-10006	1-2202|7020-7118|7202-10006	Tn4401a
CABFYH010000003.1	1	1	1	Tn4401|1|KT378596.1	Tn4401|1|KT378596.1	1-6602|7020-7118|7202-10006	1-2202|7020-7118|7202-10006	Tn4401a
MT560060.1	1	1	1	Tn4401|1|KT378596.1	Tn4401|1|KT378596.1	1-6602|7020-7118|7202-10006	1-2202|7020-7118|7202-10006	Tn4401a
CABGAI010000003.1	1	1	1	Tn4401|1|KT378596.1	Tn4401|1|KT378596.1	1-6602|7020-7118|7202-10006	1-2202|7020-7118|7202-10006	Tn4401a

Contig Name	Flanker 500bp	Flanker 5000 bp	Flanker 7200 bp	MEFinder 500bp	MEFinder 5000bp	TETyper 500bp	TETyper 5000bp	TETyper Tn4401 whole
CABGAZ010000003.1	1	1	1	Tn4401|1|KT378596.1	Tn4401|1|KT378596.1	1-6602|7020-7118|7202-10006	1-2202|7020-7118|7202-10006	Tn4401a
CABFYZ010000002.1	1	1	1	Tn4401|1|KT378596.1	Tn4401|1|KT378596.1	1-6602|7020-7118|7202-10006	1-2202|7020-7118|7202-10006	Tn4401a
CABGAQ010000009.1	1	1	1	Tn4401|1|KT378596.1	Tn4401|1|KT378596.1	1-6602|7020-7118|7202-10006	1-2202|7020-7118|7202-10006	Tn4401a
CABGBS010000004.1	1	1	1	Tn4401|1|KT378596.1	Tn4401|1|KT378596.1	1-6602|7020-7118|7202-10006	1-2202|7020-7118|7202-10006	Tn4401a

Contig Name	Flanker 500bp	Flanker 5000 bp	Flanker 7200 bp	MEFinder 500bp	MEFinder 5000bp	TETyper 500bp	TETyper 5000bp	TETyper Tn4401 whole
CABFYU010000005.1	1	1	1	Tn4401|1|KT378596.1	Tn4401|1|KT378596.1	1-6602|7020-7118|7202-10006	1-2202|7020-7118|7202-10006	Tn4401a
CABGAR010000003.1	1	1	1	Tn4401|1|KT378596.1	Tn4401|1|KT378596.1	1-6602|7020-7118|7202-10006	1-2202|7020-7118|7202-10006	Tn4401a
MT560075.1	1	1	1	Tn4401|1|KT378596.1	Tn4401|1|KT378596.1	1-6602|7020-7118|7202-10006	1-2202|7020-7118|7202-10006	Tn4401a
CABFZA010000002.1	1	1	1	Tn4401|1|KT378596.1	Tn4401|1|KT378596.1	1-6602|7020-7118|7202-10006	1-2202|7020-7118|7202-10006	Tn4401a

Contig Name	Flanker 500bp	Flanker 5000 bp	Flanker 7200 bp	MEFinder 500bp	MEFinder 5000bp	TETyper 500bp	TETyper 5000bp	TETyper Tn4401 whole
CABFXY010000002.1	1	1	1	Tn4401|1|KT378596.1	Tn4401|1|KT378596.1	1-6602|7020-7118|7202-10006	1-2202|7020-7118|7202-10006	Tn4401a
CABFZG010000005.1	1	1	1	Tn4401|1|KT378596.1	Tn4401|1|KT378596.1	1-6602|7020-7118|7202-10006	1-2202|7020-7118|7202-10006	Tn4401a
CABFYK010000005.1	1	1	1	Tn4401|1|KT378596.1	Tn4401|1|KT378596.1	1-6602|7020-7118|7202-10006	1-2202|7020-7118|7202-10006	Tn4401a
CABFXS010000003.1	1	1	1	Tn4401|1|KT378596.1	Tn4401|1|KT378596.1	1-6602|7020-7118|7202-10006	1-2202|7020-7118|7202-10006	Tn4401a

Contig Name	Flanker 500bp	Flanker 5000bp	Flanker 7200bp	MEFinder 500bp	MEFinder 5000bp	TETyper 500bp	TETyper 5000bp	TETyper Tn4401 whole
CABFYY010000002.1	1	1	1	Tn4401|1|KT378596.1	Tn4401|1|KT378596.1	1-6602|7020-7118|7202-10006	1-2202|7020-7118|7202-10006	Tn4401a
CABGAY010000002.1	1	1	1	Tn4401|1|KT378596.1	Tn4401|1|KT378596.1	1-6602|7020-7118|7202-10006	1-2202|7020-7118|7202-10006	Tn4401a
CABGBH010000002.1	1	1	1	Tn4401|1|KT378596.1	Tn4401|1|KT378596.1	1-6602|7020-7118|7202-10006	1-2202|7020-7118|7202-10006	Tn4401a
CABFYC010000004.1	1	1	1	Tn4401|1|KT378596.1	Tn4401|1|KT378596.1	1-6602|7020-7118|7202-10006	1-2202|7020-7118|7202-10006	Tn4401a

Contig Name	Flanker 500bp	Flanker 5000 bp	Flanker 7200 bp	MEFinder 500bp	MEFinder 5000bp	TETyper 500bp	TETyper 5000bp	TETyper Tn4401 whole
CABFYE010000002.1	1	1	1	Tn4401|1|KT378596.1	Tn4401|1|KT378596.1	1-6602|7020-7118|7202-10006	1-2202|7020-7118|7202-10006	Tn4401a
CABGAK010000004.1	1	1	1	Tn4401|1|KT378596.1	Tn4401|1|KT378596.1	1-6602|7020-7118|7202-10006	1-2202|7020-7118|7202-10006	Tn4401a
CABFYG010000003.1	1	1	1	Tn4401|1|KT378596.1	Tn4401|1|KT378596.1	1-6602|7020-7118|7202-10006	1-2202|7020-7118|7202-10006	Tn4401a
CABGAX010000009.1	1	1	1	Tn4401|1|KT378596.1	Tn4401|1|KT378596.1	1-6602|7020-7118|7202-10006	1-2203|7020-7118|7202-10006	Tn4401a

Contig Name	Flanker 500bp	Flanker 5000bp	Flanker 7200bp	MEFinder 500bp	MEFinder 5000bp	TETyper 500bp	TETyper 5000bp	TETyper Tn4401 whole
CABFXR010000002.1	1	1	1	Tn4401|1|KT378596.1	Tn4401|1|KT378596.1	1-6602|7020-7118|7202-10006	1-2202|7020-7118|7202-10006	Tn4401a
CABFYB010000005.1	1	1	1	Tn4401|1|KT378596.1	Tn4401|1|KT378596.1	1-6602|7020-7118|7202-10006	1-2202|7020-7118|7202-10006	Tn4401a
CABFYS010000003.1	1	1	1	Tn4401|1|KT378596.1	Tn4401|1|KT378596.1	1-6602|7020-7118|7202-10006	1-2202|7020-7118|7202-10006	Tn4401a
CABFYL010000004.1	1	1	1	Tn4401|1|KT378596.1	Tn4401|1|KT378596.1	1-6602|7020-7118|7202-10006	1-2202|7020-7118|7202-10006	Tn4401a

Contig Name	Flanker 500bp	Flanker 5000 bp	Flanker 7200 bp	MEFinder 500bp	MEFinder 5000bp	TETyper 500bp	TETyper 5000bp	TETyper Tn4401 whole
CABFYT010000008.1	1	1	1	Tn4401|1|KT378596.1	Tn4401|1|KT378596.1	1-6602|7020-7118|7202-10006	1-2202|7020-7118|7202-10006	Tn4401a
MT560078.1	1	1	1	Tn4401|1|KT378596.1	Tn4401|1|KT378596.1	1-6602|7020-7118|7202-10006	1-2202|7020-7118|7202-10006	Tn4401a
CABGBM010000006.1	1	1	1	Tn4401|1|KT378596.1	Tn4401|1|KT378596.1	1-6602|7020-7118|7202-10006	1-2202|7020-7118|7202-10006	Tn4401a
CABFYN010000003.1	1	1	1	Tn4401|1|KT378596.1	Tn4401|1|KT378596.1	1-6602|7020-7118|7202-10006	1-2202|7020-7118|7202-10006	Tn4401a

Contig Name	Flanker 500bp	Flanker 5000 bp	Flanker 7200 bp	MEFinder 500bp	MEFinder 5000bp	TETyper 500bp	TETyper 5000bp	TETyper Tn4401 whole
CABFZZ010000003.1	1	1	1	Tn4401|1|KT378596.1	Tn4401|1|KT378596.1	1-6602|7020-7118|7202-10006	1-2202|7020-7118|7202-10006	Tn4401a
CABFZH010000004.1	1	1	1	Tn4401|1|KT378596.1	Tn4401|1|KT378596.1	1-6602|7020-7118|7202-10006	1-2202|7020-7118|7202-10006	Tn4401a
CABFYM010000002.1	1	1	1	Tn4401|1|KT378596.1	Tn4401|1|KT378596.1	1-6602|7020-7118|7202-10006	1-2202|7020-7118|7202-10006	Tn4401a
MT560080.1	2	4	5	No hits	Tn1000|1|X60200.1	1-7127|7202-10006	1-7127|7202-10006	unknown

Contig Name	Flanker 500bp	Flanker 5000 bp	Flanker 7200 bp	MEFinder 500bp	MEFinder 5000bp	TETyper 500bp	TETyper 5000bp	TETyper Tn4401 whole
MT560061.1	2	4	4	No hits	Tn1000|1|X60200.1	1-7127|7202-10006	1-7127|7202-10006	unknown
MT560063.1	2	3	6	No hits	Tn1000|1|X60200.1	1-7127|7202-10006	1-7127|7202-10006	unknown
CABFYR010000005.1	3	2	2	No hits	Tn6296|1|FJ628167	1-7127|7202-10006	1-7127|7202-10006	Tn4401_truncC
CABGBR010000009.1	3	2	2	No hits	Tn6296|1|FJ628167	1-7127|7202-10006	1-7127|7202-10006	Tn4401_truncC
MT560066.1	3	5	3	No hits	No hits	1-7127|7202-10006	1-7127|7202-10006	Tn4401_truncC
CABFYJ010000003.1	4	1	8	Tn4401|1|KT378596.1	Tn4401|1|KT378596.1	1-6633|70	1-2233|70	unknown

Contig Name	Flanker 500bp	Flanker 5000 bp	Flanker 7200 bp	MEFinder 500bp	MEFinder 5000bp	TETyper 500bp	TETyper 5000bp	TETyper Tn4401 whole
						08-7075|7202-10006	08-7075|7202-10006	
CABFZC010000002.1	4	1	7	Tn4401|1|KT378596.1	Tn4401|1|KT378596.1	1-6633|7008-7075|7202-10006	1-2233|7008-7075|7202-10006	Tn4401d

Flank Pattern	N
FP1	1
FP2	37
FP3	8
FP4	0
FP5	4
FP6	230

Flank Pattern	N
FP7	2
FP8	0
FP9	5
FP10	1
FP11	4
FP12	2
FP13	9
FP14	1
FP15	13
FP16	108
FP17	0

Table S3 – Number of bla_{OXA-48} Flank Patterns (FPs) observed in the EuSCAPE short read assembly dataset. For each FP identified by Flanker in the hybrid assembly dataset (2200bp upstream of the gene), one random representative was chosen (see main methods). EuSCAPE short read assemblies (n=425 total) were screened for containment of these using mash and the FP was taken to be the match with greatest containment.

	Group 1	Group 2	Group 7	Group 8
Austria	0	0	1	0
Belgium	0	0	0	0

	Group 1	Group 2	Group 7	Group 8
Croatia	0	0	0	0
France	0	0	0	0
Germany	0	0	2	1
Greece	0	0	0	3
Ireland	0	0	0	0
Israel	0	0	19	0
Italy	0	0	1	0
Luxembourg	0	0	0	0
Macedonia	0	0	0	0
Poland	0	0	2	0
Portugal	0	0	34	0
Romania	0	0	0	0
Slovakia	0	0	0	0
Spain	0	5	0	0
United Kingdom (England, Wales & N. Ireland)	0	0	0	2

Table S4 – Geographical distribution of $bla_{KPC-2/3}$ flanking patterns observed in the EuSCAPE short read assembly dataset (n=313 with linkable geographic data). For each FP identified by Flanker in the hybrid assembly dataset (7200bp upstream of the gene), one random representative was chosen (see main methods). EuSCAPE short read assemblies were screened for containment of these using mash and the FP was taken to be the match with greatest containment. There were no isolates assigned to FPs3/4/5/6 that had linkable geographic data.

Meropenem MIC (mg/L)	FP 1	FP 2	FP 7	FP 8
≤0.06	0	0	0	0
0.12	0	1	0	0
0.25	0	0	0	0
0.5	0	0	0	0
1	0	0	0	0
2	0	1	1	0
4	0	1	1	0
8	0	1	0	1
16	0	0	3	3
32	0	0	6	1
>32	0	1	14	1

Table S5 – Distribution of meropenem minimum inhibitory concentrations (MIC) by blaKPC-2/3 7200bp upstream flanking region in the EuSCAPE dataset (n= 274 with linkable phenotypic data). For each FP identified by Flanker in the hybrid assembly dataset (7200bp upstream of the gene), one random representative was chosen (see main methods). EuSCAPE short read assemblies were screened for containment of these using mash and the FP was taken to be the match with greatest containment. There were no isolates assigned to FPs3/4/5/6 that had linkable phenotypic data.
